# Macrophage Migration Inhibitory Factor Mediates PAR-Induced Bladder Pain

**DOI:** 10.1371/journal.pone.0127628

**Published:** 2015-05-28

**Authors:** Dimitrios E. Kouzoukas, Katherine L. Meyer-Siegler, Fei Ma, Karin N. Westlund, David E. Hunt, Pedro L. Vera

**Affiliations:** 1 Research and Development, Lexington Veterans Affairs Medical Center, Lexington, Kentucky, United States of America; 2 Department of Natural Sciences, St. Petersburg College, St. Petersburg, Florida, United States of America; 3 Department of Physiology, University of Kentucky, Lexington, Kentucky, United States of America; 4 Department of Surgery, University of Kentucky, Lexington, Kentucky, United States of America; 5 Saha Cardiovascular Research Center, University of Kentucky, Lexington, Kentucky, United States of America; Northwestern University, UNITED STATES

## Abstract

**Introduction:**

Macrophage migration inhibitory factor (MIF), a pro-inflammatory cytokine, is constitutively expressed in urothelial cells that also express protease-activated receptors (PAR). Urothelial PAR1 receptors were shown to mediate bladder inflammation. We showed that PAR1 and PAR4 activator, thrombin, also mediates urothelial MIF release. We hypothesized that stimulation of urothelial PAR1 or PAR4 receptors elicits release of urothelial MIF that acts on MIF receptors in the urothelium to mediate bladder inflammation and pain. Thus, we examined the effect of activation of specific bladder PAR receptors on MIF release, bladder pain, micturition and histological changes.

**Methods:**

MIF release was measured *in vitro* after exposing immortalized human urothelial cells (UROtsa) to PAR1 or PAR4 activating peptides (AP). Female C57BL/6 mice received intravesical PAR1- or PAR4-AP for one hour to determine: 1) bladder MIF release *in vivo* within one hour; 2) abdominal hypersensitivity (allodynia) to von Frey filament stimulation 24 hours after treatment; 3) micturition parameters 24 hours after treatment; 4) histological changes in the bladder as a result of treatment; 5) changes in expression of bladder MIF and MIF receptors using real-time RT-PCR; 6) changes in urothelial MIF and MIF receptor, CXCR4, protein levels using quantitative immunofluorescence; 7) effect of MIF or CXCR4 antagonism.

**Results:**

PAR1- or PAR4-AP triggered MIF release from both human urothelial cells *in vitro* and mouse urothelium *in vivo*. Twenty-four hours after intravesical PAR1- or PAR4-AP, we observed abdominal hypersensitivity in mice without changes in micturition or bladder histology. PAR4-AP was more effective and also increased expression of bladder MIF and urothelium MIF receptor, CXCR4. Bladder CXCR4 localized to the urothelium. Antagonizing MIF with ISO-1 eliminated PAR4- and reduced PAR1-induced hypersensitivity, while antagonizing CXCR4 with AMD3100 only partially prevented PAR4-induced hypersensitivity.

**Conclusions:**

Bladder PAR activation elicits urothelial MIF release and urothelial MIF receptor signaling at least partly through CXCR4 to result in abdominal hypersensitivity without overt bladder inflammation. PAR-induced bladder pain may represent an interesting pre-clinical model of Interstitial Cystitis/Painful Bladder Syndrome (IC/PBS) where pain occurs without apparent bladder injury or pathology. MIF is potentially a novel therapeutic target for bladder pain in IC/PBS patients.

## Introduction

Macrophage migration inhibitory factor (MIF) is a widely-expressed and versatile cytokine that plays key roles in several pain [1, 2] and inflammatory diseases [3, 4], including neuropathic pain, rheumatoid arthritis, atherosclerosis, asthma, sepsis, and bladder inflammation [1, 2, 5–10]. In the bladder, urothelial cells constitutively express and store MIF in intracellular pools that are rapidly released upon inflammatory stimulation [9, 11–13]. Released intraluminal MIF then activates urothelial MIF receptors to further mediate other inflammatory changes and pain in the bladder [7, 8, 14, 15].

Protease-activated receptors (PARs) mediate signal transduction when activated proteases cleave the tethered ligand in the extracellular domain in the receptor thus freeing it to bind to the receptor complex [16]. Urothelial cells express all four PAR receptors (PAR1, PAR2, PAR3, PAR4) [17, 18], and we reported that human and rat urothelial cells constitutively express MIF and express PAR1 [15]. Intravesical PAR receptor stimulation resulted in bladder inflammation as measured by histology [19] and expression of inflammatory cytokines [20]. Physiological effects of intravesical PAR stimulation, such as bladder pain and/or micturition parameters, have not been investigated. We previously reported that intravesical thrombin (presumably through activation of urothelial PAR1 receptors) also triggered urothelial MIF release from both human urothelial cells in culture and rat bladders *in vivo* [15]. Therefore, we hypothesized that intravesical stimulation of specific PAR receptors results in intraluminal MIF release that then activates MIF urothelial receptors to mediate inflammatory changes and pain in the bladder.

In this study, we tested the hypothesis that specific activation of PAR1 or PAR4 bladder receptors results in urothelial MIF release and in turn MIF-mediated signaling that produces bladder pain and inflammation. Studies using human (SV40-transformed) urothelial cells (UROtsa) examined dose-effect and time-course of *in vitro* MIF release from PAR1 or PAR4 stimulation. Additional studies on female mice were performed by intravesical instillation of PAR1 or PAR4 activating peptides (AP) to determine: 1) urothelial MIF release; 2) abdominal sensitivity to von Frey filament stimulation twenty-four hours after AP exposure as a measure of bladder pain; 3) awake micturition changes twenty-four hours after AP; 4) changes in bladder histology due to treatment; 5) changes in bladder levels of MIF and MIF receptors using real-time RT-PCR and immunofluorescence; and 6) effect of pharmacological blockade of MIF or MIF receptors.

## Methods

### *In Vitro* Experiments

Human SV40-transformed urothelial cells (UROtsa; a kind gift of Scott H. Garrett [21]) were plated in 24-well plates with 5 replicates per treatment group at a density of 6 x 10^4^ cells/ml overnight in DMEM with 10% FBS. Cells were synchronized one hour in fresh DMEM (0.1% BSA) before exchanging this medium for DMEM (0.1% BSA) containing a human PAR activating peptide (PAR1-AP = TFLLR-NH2; PAR4-AP = AYPGKF-NH2) or a corresponding scrambled control peptide (PAR1 control = RLLFT-NH2; PAR4 control = YAPGKF-NH2) at either 25 or 50 μM (Peptides International, Inc.; Louisville, KY). Cultured medium was collected at 15, 60, and 120 minutes and assayed for MIF by ELISA (R&D Systems; Minneapolis, MN).

### *In Vivo* Experiments

All animal experiments were approved by Lexington Veterans Affairs Medical Center Institutional Animal Care and Use Committee (VER-11-016-HAF). Procedures were carried out humanely to minimize suffering and were performed according to the guidelines of the National Institutes of Health. For survival studies, mice were checked after instillation and at the end of the day for signs of urethral bleeding or extreme discomfort (end-point criteria for euthanasia). No mice were observed to meet euthanasia criteria. No post-surgical analgesia was used as this may have reduced pain related behaviors.

#### Mechanical Allodynia

Thirteen week-old female mice (C57BL/6; Jackson Laboratory, Bar Harbor, ME) were acclimated to the procedure room and experimenters over four separate 15 minute sessions (1 session/day) before measuring mechanical allodynia. Mice were placed individually in clear plastic boxes (56 x 39 x 38 mm) on an elevated metal mesh screen [22] and a von Frey filament (0.008 g bending force) was pressed to the lower abdominal / perineal area of each mouse five times during each acclimation session. After the final acclimation session and 24 hours before baseline testing, the lower abdominal region was shaved under isoflurane anesthesia. Von Frey filaments of ascending bending force (0.008, 0.020, 0.040, 0.070 g) were applied in trials of 10 [23] to assess baseline responses to abdominal / perineal stimulation prior to instillation of PAR activating peptides. Positive responses consisted of 1) licking the application area, 2) flinching/jumping, 3) or abdomen withdrawal. Mice responding more than 30% to the weakest filament (0.008 g) during baseline testing were excluded from the study. This procedure was repeated 24 hours after intravesical PAR-AP instillation.

#### Intravesical Instillation of PAR Peptides

Mice were anesthetized with isoflurane and transurethrally catheterized (PE10, 11 mm length). Urine was drained by gently applying pressure to the lower abdomen [19], and bladders were slowly instilled with solutions of either a PAR-activating peptide [PAR1-AP (TFLLR-NH2), or PAR4-AP (AYPGKF-NH2)], or a corresponding scrambled control peptide [PAR1 control (RLLFT-NH2), or PAR4 control (YAPGKF-NH2)]. The peptides (100 μM) in sterile phosphate-buffered saline diluent (PBS, pH 7.4, 150 μl) were retained in the bladder for 1 hour. The intraluminal fluid (ILF) was collected from the catheter tip, treated with protease inhibitors (Halt III; Thermo Sci., Rockford, IL), and stored at -80°C until testing.

In survival experiments, mice also received (i.p.) pre-treatment with either MIF antagonist, ISO-1 (20 mg/kg, 20% DMSO in saline; Calbiochem, Billerica, MA), CXCR4 antagonist, AMD3100 (10 mg/kg in saline; Tocris, Minneapolis, MN), or a vehicle control 15 minutes before the instillation of PAR-activating peptides as indicated in Table 1. ILF was collected as described above.

**Table 1:** Treatment groups for experiments.

Group	N	Pretreatment (i.p.)	Intravesical
Veh + Control Pep	8	vehicle	scrambled control
Veh + PAR1-AP	12	vehicle	PAR1-AP
ISO-1 + PAR1-AP	8	ISO-1	PAR1-AP
Veh + PAR4-AP	8	vehicle	PAR4-AP
ISO-1 + PAR4-AP	8	ISO-1	PAR4-AP
AMD3100 + PAR4-AP	8	AMD3100	PAR4-AP

Mice received a pre-treatment (i.p.) of vehicle (Veh: 20% DMSO in saline), MIF antagonist (ISO-1: 20 mg/kg, 20% DMSO in saline), or CXCR4 antagonist (AMD3100: 10 mg/kg in saline) 15 minutes before intravesical instillation of a scrambled peptide (Control Pep), PAR1-AP, or PAR-4AP (all 100 μM).

#### Micturition

Twenty four hours after intravesical peptide administration, awake micturition volume and frequency were determined using the voided stain on paper method (VSOP) [24] as reported earlier [7]. Briefly, mice were gavage-fed drinking water (50 μl/g body weight) and placed in clear cages over a wire mesh elevated above filter paper for 2 hours. Each micturition was collected on filter paper below. Voided volumes were estimated by comparing stain areas on filter paper against measured volumes through linear regression, while micturition frequency was defined as total number of urinations in two hours.

#### Bladder Tissue Collection

After PAR-AP infusion for 1 hour or von Frey/VSOP testing 24 hour after activating peptide instillation, bladders were removed under isoflurane anesthesia before euthanizing mice by thoracotomy. Bladders were sectioned in half longitudinally and bladder portions were frozen on dry ice for protein or RNA processing, or fixed in 10% formalin.

#### Histology and Immunohistochemistry

Transverse pieces of fixed bladder from the mid-detrusor region were embedded in paraffin for routine hematoxylin and eosin (H&E) staining or immunohistochemistry. H&E stained sections (5 μm) were evaluated by a pathologist blinded to the experimental treatment and scored for edema and inflammation.

For immunohistochemistry, bladder paraffin sections (5 μm; N = 6/group; randomly selected) were dewaxed and exposed to 10 mM citrate buffer (pH 6.0; 94 C; 40 min) for antigen unmasking. Bladder sections were blocked (5% goat serum, 0.2% Triton X-100 in PBS) for 30 minutes at room temperature and incubated overnight at 4°C with rabbit anti-MIF antibody (Torrey Pines Biolabs; Secaucus, NJ; 1:50 in PBS with 1% goat serum, 0.2% Triton X-100) or rabbit anti-CXCR4 antibody (Sigma-Aldrich; St. Louis, MO; 1:50 in PBS with 1% goat serum, 0.2% Triton X-100). Sections were exposed to goat anti-rabbit TRITC-labeled secondary antibody (Jackson Laboratory; Bar Harbor, ME; 1:50 in PBS with 1% goat serum, 0.2% Triton X-100) for one hour at room temperature and cover-slipped with vectashield mounting medium (Vector Laboratories; Burlingame, CA) to minimize photobleaching. In control slides, primary antisera were omitted but were otherwise processed as above. Immunofluorescence was visualized using a Leica DMI 4000B microscope (Wetzlar, Germany) and digital images were obtained (Leica Application Software v4.5; Wezlar, Germany).

Quantitative immunohistochemistry image analysis was performed in ImageJ (NIH, Washington DC). For each bladder, intensity values represent an average of mean grey values recorded from region-of-interests (ROIs) drawn around the urothelium in three view-fields of the same section.

#### MIF ELISA

MIF levels in UROtsa culture media were measured with a commercial ELISA kit (DuoSet human MIF ELISA; R&D Systems; Minneapolis, MN). Mice intraluminal fluid (ILF) MIF levels were assessed using a custom ELISA protocol (modified for small volumes) for detecting mouse MIF [8] and normalized to creatinine (Crystal Chem, Inc.; Downers Grove, IL). MIF levels were estimated using 4-point parameter log-logistic regression in SoftMax Pro (Molecular Devices; Sunnyvale, CA). All assays were performed in duplicate.

#### Real-Time RT-PCR

RNA from bladder tissues was extracted and converted to cDNA using the TRIzol method (Invitrogen; Grand Island, NY) and commercial reverse transcription system (Reverse Transcription System; Promega; Madison, WI) as previously described [15]. Sequences for MIF and MIF receptors, CD74, CXCR2 and CXCR4, were amplified with specific primers and RT^2^ SYBR green real-time RT-PCR master mix (Qiagen; Valencia, CA) according to manufacturer’s instructions. Thermal cycling in a StepOnePlus Real-Time PCR System (Applied Biosystems; Grand Island, NY) proceeded as follows: 10 minutes at 95°C, followed by 40 cycles of denaturation (15 seconds at 95°C) and annealing / extension (60 seconds at 60°C). Fold changes were determined using the ΔΔCT method with 18S rRNA as the reference housekeeping gene. Data are from duplicate wells of each bladder sample.

### Statistical Analysis

For *in vitro* experiments, culture media MIF levels from UROtsa cultures receiving PAR-AP were compared against those exposed to respective control peptides in planned comparisons (Student’s t-tests). *In vivo* differences in intraluminal MIF levels and micturition parameters were assessed in planned comparisons (Student’s t-tests). Mann-Whitney U analyses determined significant differences in fold expression (ΔCT). Significant changes in abdominal hypersensitivity were examined using paired t-tests comparing positive response frequency (%) at baseline to that at 24 hours after treatment. Differences in immunofluorescence intensity were assessed in planned comparisons (Student’s t-tests).

All data are presented as mean ± SE. Statistical differences of *p* ≤ 0.05 were considered significant. All statistical analyses were performed using SPSS Statistics (IBM; New York, NY).

## Results

### PAR1 or PAR4 activation induced MIF release from urothelium

**Fig 1:**
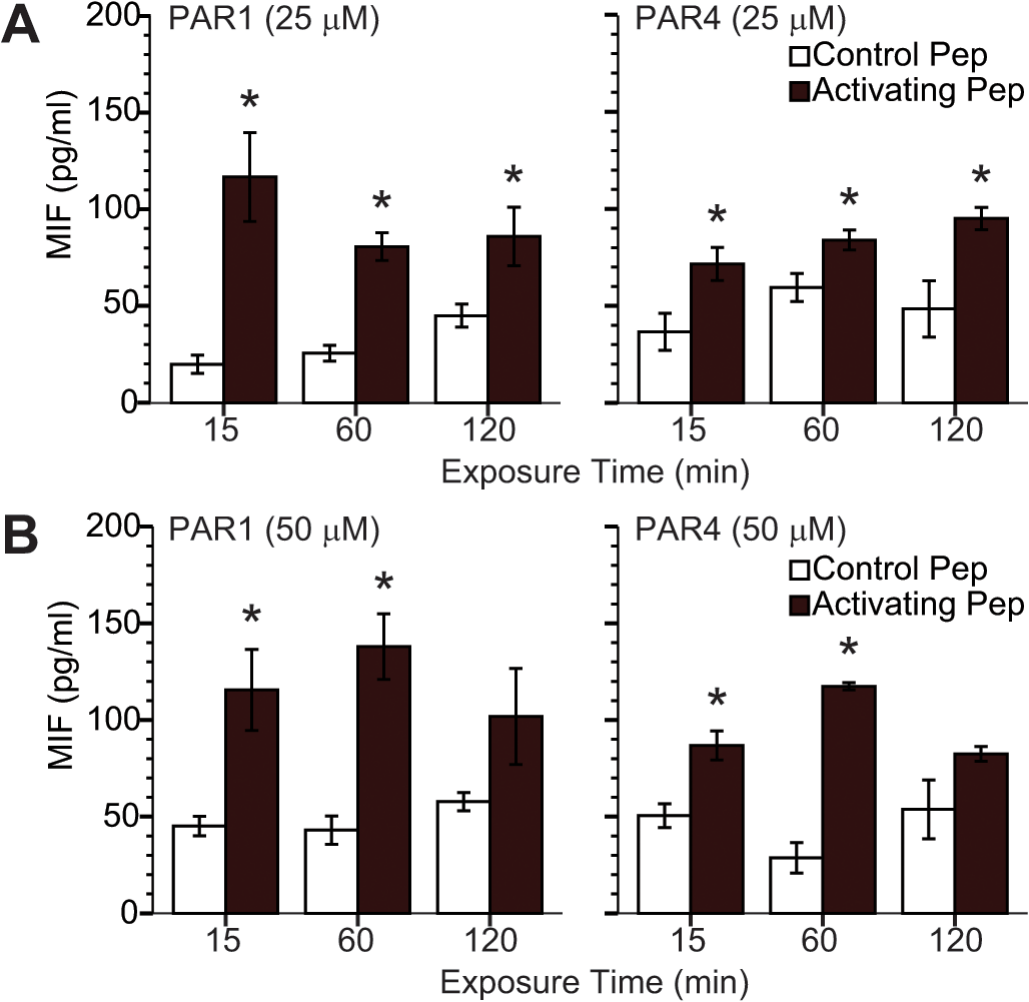
Human urothelial cultures release MIF after activating PAR1 or PAR4. Human benign transformed urothelial cells (UROtsa) were exposed to specific human PAR1 (TFLLR-NH2) or PAR4 (AYPGKF-NH2) activating peptides (AP), or respective scrambled peptides (control). Culture media was collected at 15, 60, and 120 minutes after AP application and assayed for MIF using a commercial ELISA kit (R&D Systems; Minneapolis, MN). Significant urothelial MIF release occurring within the first 15 minutes was observed after PAR1 or PAR4 stimulation either at 25 μM (**A**) or 50 μM (**B**) concentrations. * *p* ≤ 0.05.

We previously demonstrated that thrombin, an activator of PAR1 and PAR4 receptors, elicited MIF release from transformed human urothelial cells (UROtsa) [15]. In the present study, we examined the respective contribution of PAR1 or PAR4 to human urothelial MIF release. Fig 1 shows that either PAR1-AP or PAR4-AP at concentrations of 25 μM (1A) and 50 μM (1B) induced MIF release from UROtsa cells into the culture media. For example, PAR1-AP (25 μM for 15 minutes) resulted in media MIF levels of 87.4 ± 17.2 pg/ml in comparison to 15.0 ± 3.5 pg/ml elicited by the inactive PAR1 control peptide (RLLFT-NH2). Furthermore, this MIF release occurs within 15 minutes of exposing UROtsa cultures to PAR1 or PAR4 activating peptides.

**Fig 2:**
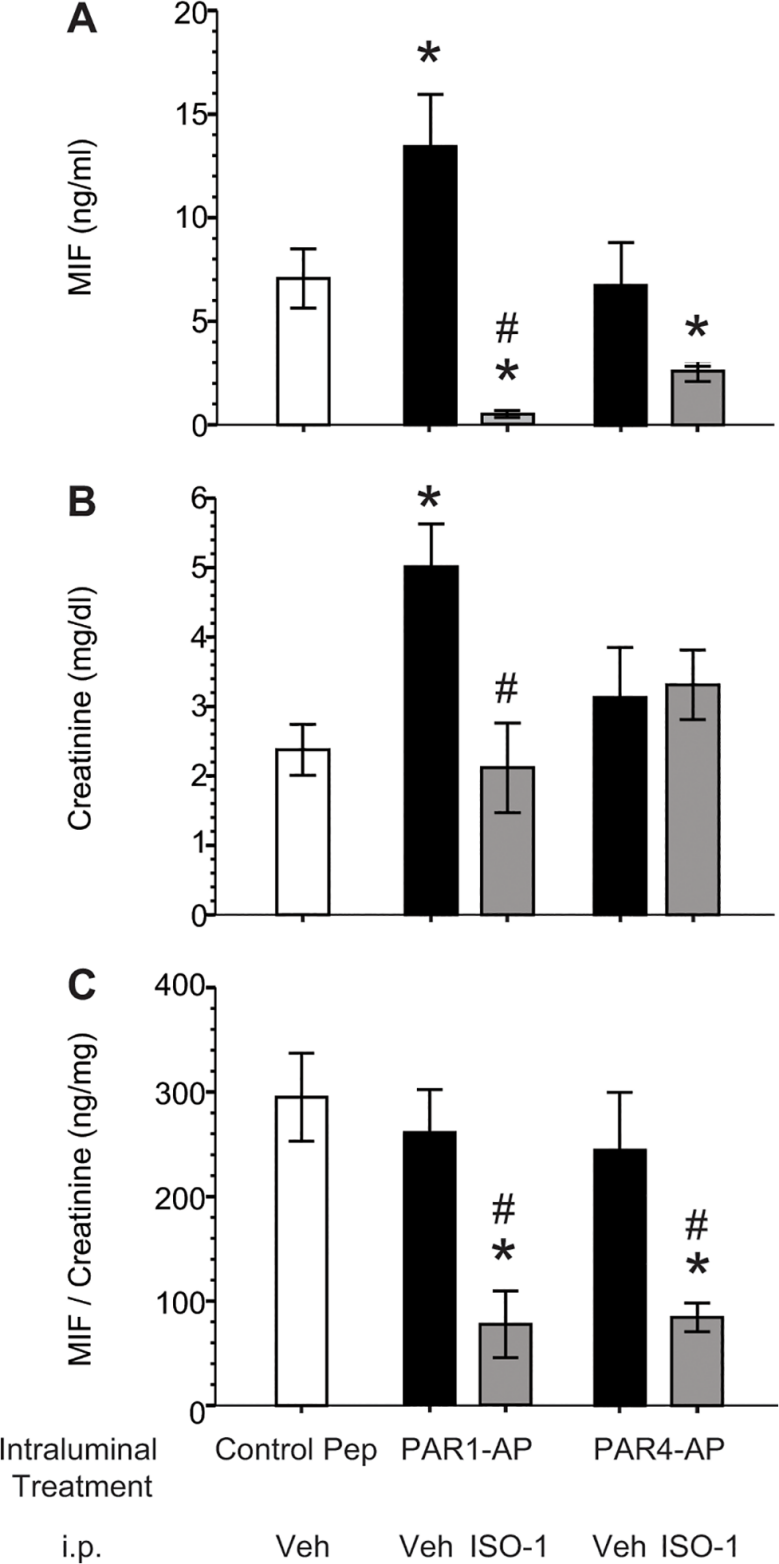
ELISA did not detect PAR1- or PAR4-evoked urothelial MIF release *in vivo*. Anesthetized female mice received intravesical instillation of a specific mouse PAR1 or PAR4 activating peptide or a scrambled peptide (control) for one hour. In addition, they received either MIF antagonist (ISO-1) or vehicle (20% DMSO) i.p. 15 minutes prior to intravesical instillation. The intraluminal fluid was collected and assayed for MIF by ELISA as previously described [11] and for creatinine concentration. **A**) MIF concentration (pg/ml) in the intraluminal fluid was increased after PAR1, but not PAR4 stimulation, relative to control (scrambled) peptide stimulation. This effect was prevented by MIF inhibition through ISO-1 pre-treatment. ISO-1 also decreased MIF concentration in PAR4-AP treated animals as compared to controls. **B**) Creatinine concentrations were higher in PAR1-AP treated animals but not in PAR4-AP treated animals. **C**) When MIF concentrations were normalized to creatinine, PAR stimulation (with vehicle pre-treatment) did not show a difference when compared to controls (vehicle pre-treatment; scrambled peptide). ISO-1 pre-treatment significantly decreased ng MIF/mg Creatinine in either PAR1 or PAR4 treated groups. * *p* ≤ 0.05 compared to control group (vehicle pre-treatment; intravesical scrambled peptide); # *p* ≤ 0.05 compared to vehicle pre-treatment with respective PAR-AP.

We also examined the effect of intravesical PAR1-AP or PAR4-AP administration on MIF release *in vivo* from the mouse bladder (Fig 2). MIF levels in the ILF one hour after intraluminal exposure to PAR1-AP, but not PAR4-AP, were significantly higher than those seen in mice receiving control peptide (YAPGKF-NH2; Fig 2A). However, since creatinine levels were higher in the PAR1-AP treated group (Fig 2B), normalization of MIF levels to creatinine levels to account for urine MIF, showed no difference in MIF levels after either PAR1-AP or PAR4-AP treatment (Fig 2C).

**Fig 3:**
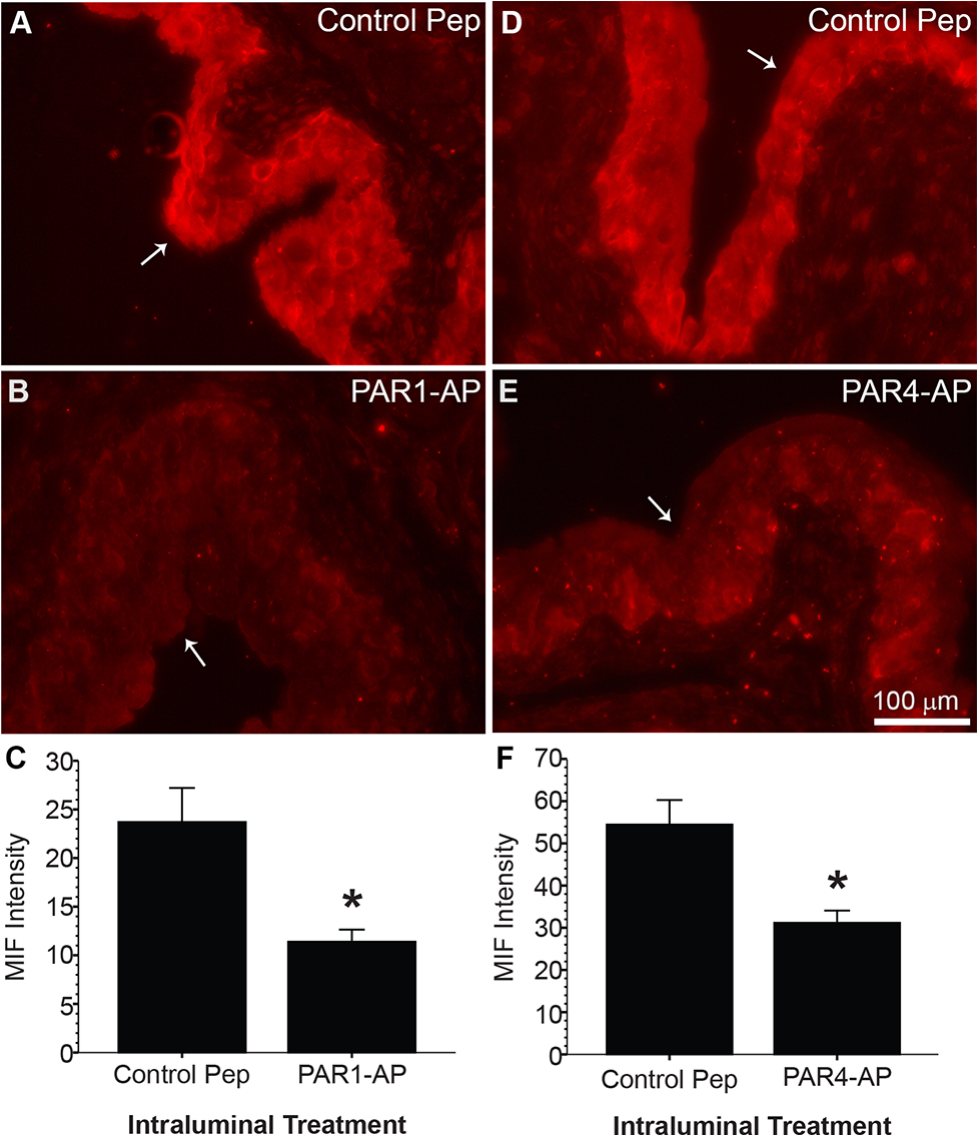
PAR1 or PAR4 activation reduces urothelial MIF in mouse bladder. Female mice under anesthesia, received intravesical instillation of a PAR1 or PAR4 activating peptide (AP) or a respective scrambled peptide (Control Pep: RLLFT-NH2 for PAR1; YAPGKF-NH2 for PAR4) for one hour before harvesting bladders. Panels (**A**, **B**, **D**, **E**) show MIF immunofluorescent labeling in representative bladders. White arrows point to intraluminal surface of the urothelia. MIF immunofluorescent labeling localized to urothelium (**A, D**) and a decrease in urothelial MIF immunofluorescence was observed after PAR1-AP (**B**) or PAR-4-AP (**E**) treatment when compared to control peptide-treated groups (**A**, **D**). Densitometry showed that average urothelial MIF immunofluorescence (N = 6 per group) significantly decreased after intravesical PAR1- (**C**) or PAR4-AP (**F**) treatment when compared to respective control groups (* *p* ≤ 0.05).

Immunofluorescence showed that MIF is located primarily in the urothelium (Fig 3A and 3D) as we also previously reported in rats [10, 15]. Densitometic analysis showed that one hour intravesical administration of PAR1-AP (Fig 3B) or PAR4-AP (Fig 3D) decreased urothelial MIF by 52 or 43% respectively, when compared to mice treated with intravesical inactive control peptide (Fig 3C and 3F; *p* ≤ 0.05). Control slides where primary antisera were omitted had no immunofluorescence (not shown).

### Activation of bladder PAR1 or PAR4 induced abdominal mechanical hypersensitivity, an effect mediated through MIF

**Fig 4:**
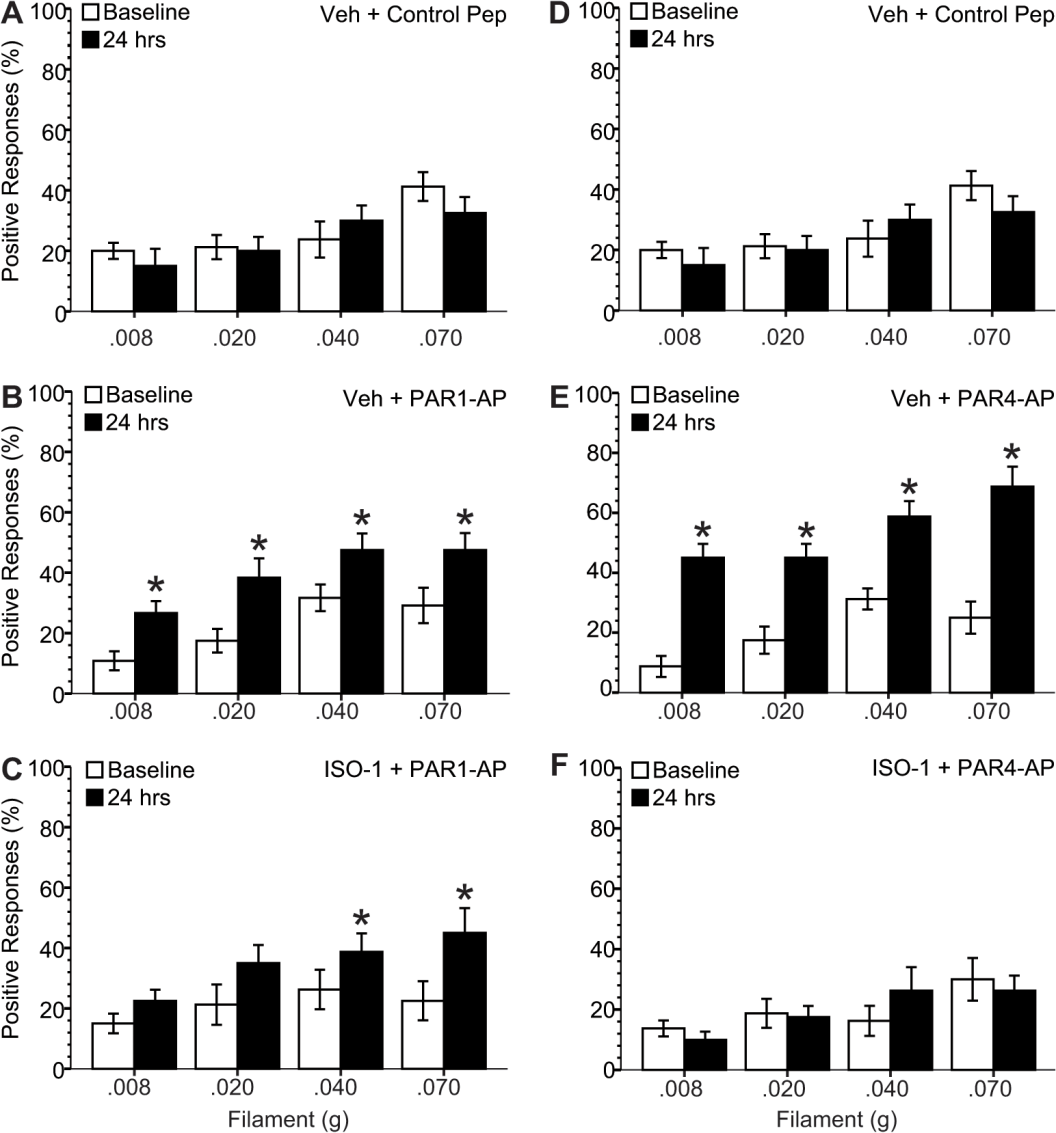
MIF mediates PAR1- or PAR4-induced mechanical hypersensitivity. Mechanical hypersensitivity was measured as % response out of 10 applications of von Frey monofilaments of graded stimulus strengths (as indicated) at baseline and 24 hour after one hour intravesical instillation of PAR-activating peptides. **A**) Control group received pre-treatment with vehicle (20% DMSO in saline; i.p.) 15 minutes before intravesical instillation of a scrambled peptide. There was no statistical difference between baseline values and 24 hours after treatment. **B**) Mice treated with intravesical PAR1-AP (and vehicle pre-treatment) showed a significant increase in the % response 24 hours after treatment at all filament strengths tested. **C**) Pre-treatment with MIF antagonist (ISO-1; 20 mg/kg, 20% DMSO in saline; i.p.) 15 minutes before intravesical PAR1-AP instillation partially prevented the baseline vs. post- 24 hour increase in response seen after PAR1-AP alone. **E**) Intravesical PAR4-AP instillation (vehicle pre-treatment) resulted in a significant increase in the % response at 24 hours when compared to baseline. **F**) This effect was completely prevented by pre-treatment with ISO-1 (MIF antagonist). * *p* ≤ 0.05 when compared to the corresponding baseline value.

We determined the effect of urothelial PAR1 or PAR4 receptor stimulation on abdominal mechanical hypersensitivity by measuring responses to von Frey filament application to the abdominal / perineal area before and 24 hours after instilling solutions containing a scrambled control peptide, PAR1-AP or PAR-4AP. No changes in mechanical threshold were detected before (baseline) and 24 hours after instillation of control peptide (Fig 4A and 4D). Twenty-four hours after PAR1-AP or PAR4-AP exposure on the other hand, there was a significantly increased response frequency to filament application (Fig 4B and 4E). For example, positive responses to the firmest filament (0.070 g) for PAR4-AP exposure increased from 25.0 ± 5.3% at baseline to 68.8 ± 6.7% at 24 hours (*p* ≤ 0.05), and for PAR1-AP increased from 29.2 ± 5.8% to 47.5 ± 5.7% (*p* ≤ 0.05) indicating a stronger effect from PAR4-AP administration. Pre-treatment with the MIF-antagonist, ISO-1, completely prevented PAR4-AP-induced hypersensitivity (Fig 4F) and reduced PAR1-AP-induced hypersensitivity (Fig 4C).

### Intravesical PAR1 or PAR4 activation did not elicit bladder inflammation

**Fig 5:**
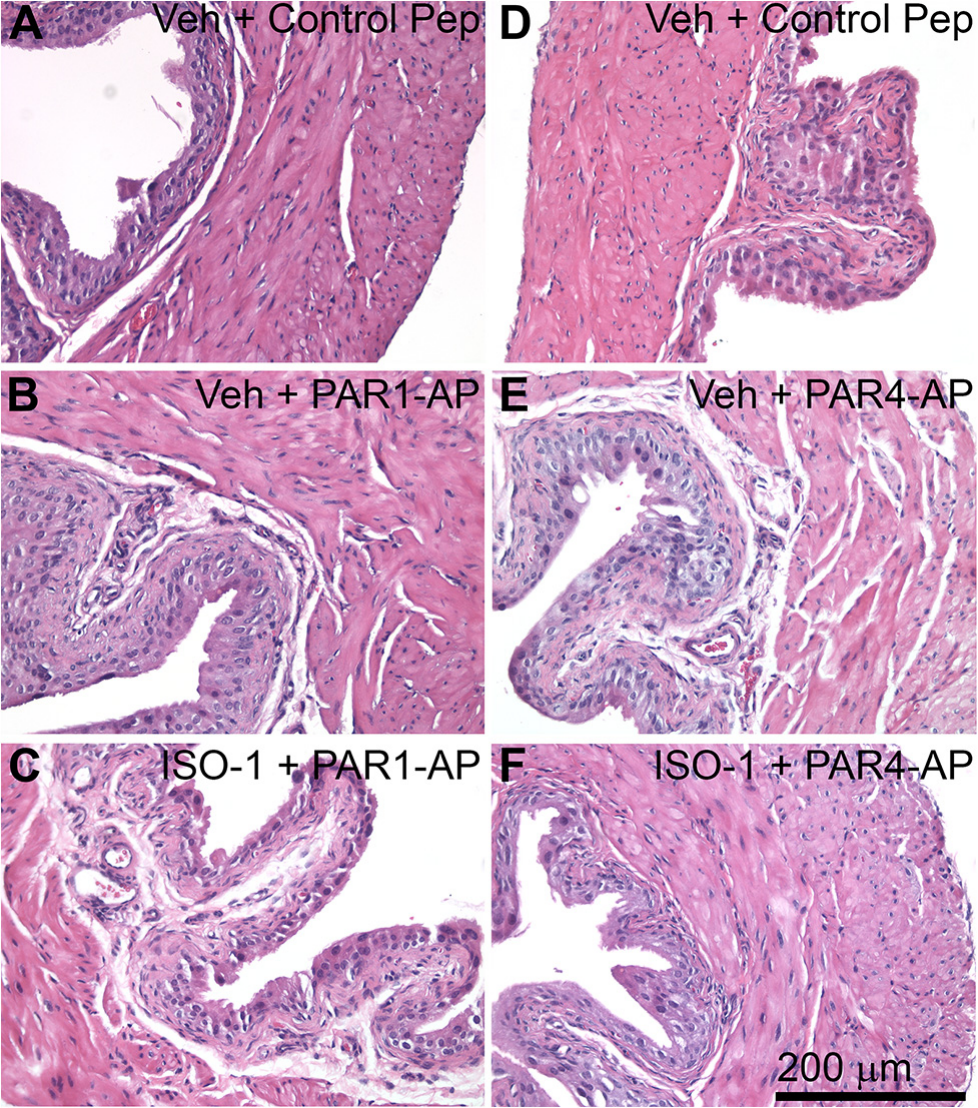
Activating urothelial PAR1 or PAR4 did not result in bladder inflammation. H&E stained paraffin bladder sections showed normal bladder and urothelial morphology in mice in the control group (**A**, **D**; vehicle pre-treatment, intravesical scrambled peptide). Intravesical treatment with PAR1-AP (**B**) or PAR4-AP (**E**) did not alter morphology. Pre-treatment with ISO-1 also had no effect (**C**, **F**).

Activation of PAR receptors was reported to result in histological signs of bladder inflammation [15, 19]. We measured bladder edema and polymorphonuclear leukocyte infiltration 24 hours after intravesical administration of PAR1-AP or PAR4-AP. No evidence of edema or polymorphonuclear leukocyte infiltration was observed after intravesical PAR1-AP or PAR4-AP administration (Fig 5B and 5E). In addition, treatment with intravesical PAR activating peptides and/or MIF antagonist, ISO-1, did not elicit physiological signs of inflammation (such as increased micturition frequency or decreased voided volumes; Table 2). Pre-treatment with ISO-1 also did not produce changes in micturition (Table 2) or bladder morphology (Fig 5C and 5F).

**Table 2:** Awake micturition parameters in PAR1-AP or PAR4-AP treated groups.

Group	N	Frequency ± s.e.	Vol. (μl) ± s.e.
Veh + Control Pep	7	3.3 ± 0.3	250.4 ± 12.2(ns)
Veh + PAR1-AP	12	3.8 ± 0.3	277.8 ± 14.1(ns)
ISO-1 + PAR1-AP	8	4.3 ± 0.6	255.1 ± 17.5(ns)
Veh + PAR4-AP	8	3 ± 0.5	275.4 ± 14.3(ns)
ISO-1 + PAR4-AP	7	3.9 ± 0.3	271.2 ± 12.3(ns)

ns = *p* > 0.05.

### PAR4 upregulated bladder CD74 and CXCR4 (MIF receptors) mRNA expression and urothelial CXCR4 levels

The urothelium constitutively expresses MIF, MIF receptors [8, 10], and PAR receptors [15, 17]. Since intravesical PAR4 induced bladder hypersensitivity that was prevented by MIF antagonism, we examined changes in mRNA expression levels of MIF and MIF receptors, CD74; CXCR2; CXCR4, in the bladder following the treatments. Real-time RT-PCR showed that intravesical PAR4-AP increased bladder CD74 and CXCR4 expression (4- and 11-fold respectively; *p* ≤ 0.05; Table 3) when compared to bladders treated with control peptide. MIF and CXCR2 expression was not significantly changed by PAR activation. Pre-treatment with MIF antagonist, ISO-1, prevented PAR-AP-induced changes in bladder CD74 and CXCR4 expression.

**Table 3:** Expression changes in bladder MIF and MIF receptors after intravesical PAR4-AP treatment.

	Veh + Control Pep	Veh + PAR4-AP	ISO-1 + PAR4-AP
MIF	1	4.14	0.99
CD74	1	4.05 *	3.15
CXCR4	1	11.01 *	0.86
CXCR2	1	2.11	3.89

* *p* ≤ 0.05.

**Fig 6:**
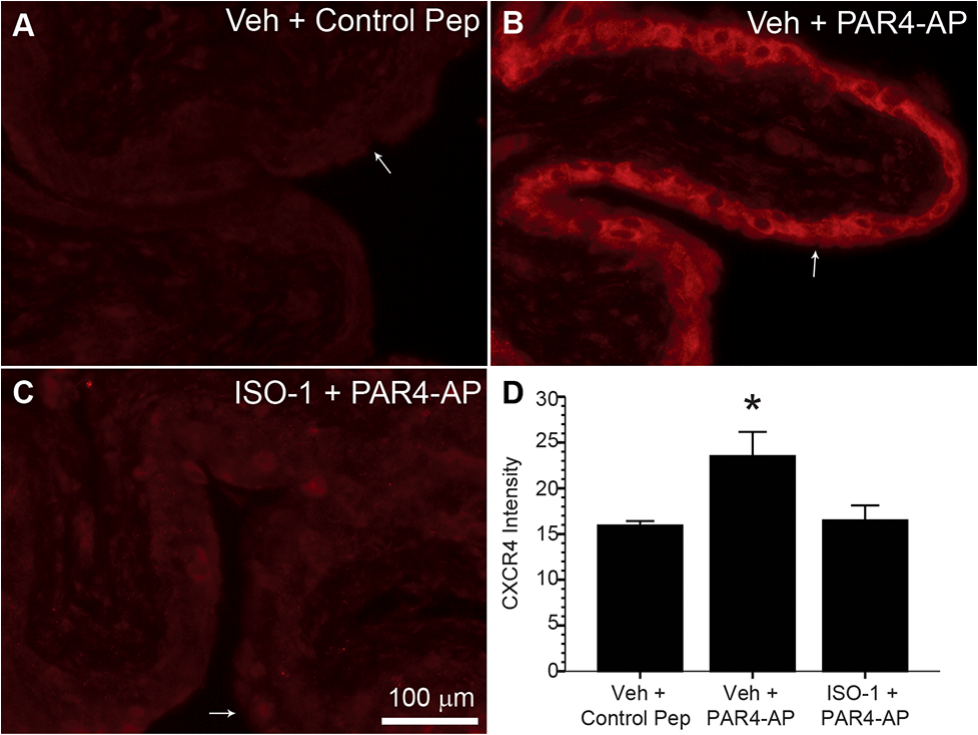
PAR4 activation increases urothelial levels of MIF receptor, CXCR4. Panels (**A**, **B**, **C**) show the CXCR4 immunofluorescence in representative bladders which is restricted to urothelium. White arrows identify the urothelium intraluminal surface. More intense urothelial CXCR4 immunofluorescent labeling is apparent in PAR-4P-exposed bladders (**B**) than in control peptide-treated bladders (**A**). Pre-treatment with MIF antagonist, ISO-1 (**C**) prevented this increase. **D**) Densitometry showed that average urothelial CXCR4 immunofluorescence (N = 6 per group) significantly increased after intravesical PAR4-AP when compared to control peptide-treated animals (* *p* ≤ 0.05). This effect was prevented by ISO-1 pre-treatment.

Since PAR4-AP exposure after 24 hours elicited a substantial increase in bladder CXCR4 mRNA, we also examined CXCR4 protein levels in the urothelium by immunofluorescence. Densitometric analysis revealed a 47.6% increase in urothelial CXCR4 in PAR4-AP-treated mice in comparison to mice treated with control peptide (*p* ≤ 0.05; Fig 6). Pre-treatment with the MIF antagonist, ISO-1, completely prevented this increase (Fig 6D).

### PAR4-induced mechanical hypersensitivity was partially reduced by CXCR4 antagonist

**Fig 7:**
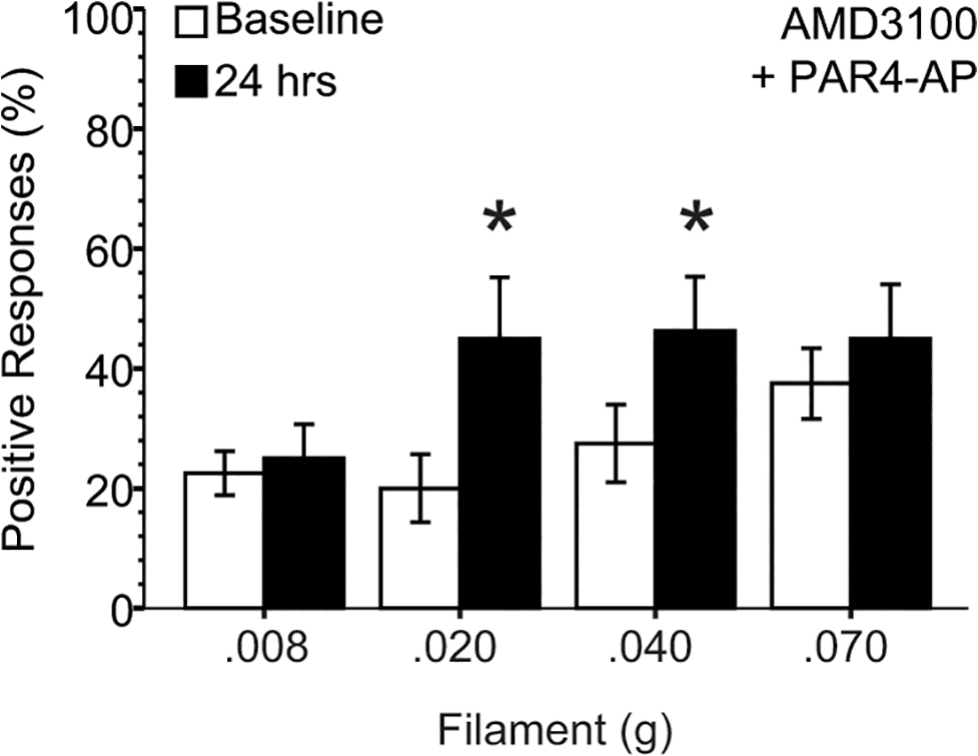
PAR4-induced mechanical hypersensitivity is partially reduced by CXCR4 antagonist, AMD3100. Intravesical PAR4-AP treatment resulted in a significant increase in % positive responses with every filament strength tested (see Fig 4E). Pre-treatment with a CXCR4 antagonist, AMD3100 (10 mg/kg; i.p.), 15 minutes before AP, reduced the PAR4-AP-induced % response increase from respective baseline (* *p* ≤ 0.05).

Given the upregulation of bladder CXCR4, we tested whether PAR4-induced mechanical hypersensitivity is mediated through MIF receptor, CXCR4. In mice pretreated with a CXCR4 antagonist, AMD3100 (10 mg/kg, i.p.), we observed partial inhibition of the PAR4-AP-induced hypersensitivity (Fig 7; compare to 4E).

## Discussion

The present study demonstrates that: 1) PAR1 or PAR4 activation released MIF from human urothelial cells and mouse urothelium; 2) intravesical installation of PAR1-AP or PAR4-AP in mice induced pain without overt bladder injury or inflammation; 4) activation of PAR4 had a greater effect than PAR1 in eliciting pain; 3) PAR-AP-induced pain was prevented by MIF antagonism indicating a role for MIF and MIF receptor CXCR4 in mediating bladder pain.

MIF is an inflammatory cytokine, highly expressed in many different types of cells, including urothelial cells [25]. In the urothelium, we showed that MIF is released quickly in response to inflammatory stimuli [12] and promotes bladder inflammation [7, 26]. Other investigators demonstrated the expression of PAR receptors in the urothelium [17] and that they are involved in mediating bladder inflammation [19, 27]. We previously showed that thrombin, a PAR1 / PAR4 receptor activator, elicits MIF release from human urothelial cells and from rat bladder [15]. Our current results, using specific PAR-activating peptides, confirm and extend our earlier findings by showing that activation of PAR1 or PAR4 induces MIF release from human urothelial cells (UROtsa). Intravesical stimulation of mouse PAR1 or PAR4 also diminished urothelial MIF levels after 1 hour, which is consistent with urothelial MIF release [10]. However, in the present study, activating with specific PAR activating peptides did not produce elevations in intraluminal MIF, unlike previously reported results using intravesical thrombin in a rat model [15].

There are several explanations for the discrepancy between our findings in rats using intravesical thrombin [15] and our current findings using specific PAR activating peptides in mice. These include: 1) different delivery methods (transurethral catheter, in this study; injection through the bladder dome, in the previous study); 2) different measures of released MIF (ng MIF/mg creatinine in this study since ureters were intact and there is MIF in urine; ng MIF/ml in the previous study where ureters were cut); 3) simultaneous activation of two receptors by thrombin in the previous study may be more effective in eliciting MIF release than single receptor activation in this study; 4) species differences.

First, urine MIF may be confounding the measurement of released urothelial MIF levels in mice with intact ureters. In our previous study in rats, we isolated the bladder by cutting both ureters prior to emptying [15]. In fact, in the present study, absolute MIF levels (in ng/ml) were increased after PAR1-AP treatment and creatinine levels differed among the groups (Fig 2A and 2B) indicating that urine protein concentrations were different. Second, it is possible that activation of more than one PAR receptor, as it is likely with thrombin, is more effective in eliciting MIF release than single PAR receptor activation. Given the robust and quick MIF release by human urothelial cells treated with either PAR1 or PAR4 activating peptides, we regard this as an unlikely possibility. Finally, rodent species differences may account for the discrepancy. We consider it likely that a different delivery route with its accompanying urine contamination may have obscured MIF release elicited by PAR activation in mice, particularly considering the clear results obtained with human urothelial cells (Fig 1). In this study, we also documented decreased urothelial MIF immunofluorescence after PAR4-AP treatment which is consistent with MIF release *in vivo* and in agreement with our previous findings [15].

The most significant effect observed in the present study was PAR-induced mechanical hypersensitivity, in the absence of histological (edema; polymorphonuclear leukocyte infiltration) or physiological (changes in micturition frequency or volume) signs of inflammation. In addition, this PAR-induced hypersensitivity was mediated by MIF, at least in part through activation of CXCR4 receptors. PAR-induced allodynia without bladder inflammation is a novel finding and significant in that we describe a manipulation that elicits bladder pain-like behaviors without overt damage to the bladder itself. Our findings are in agreement with recent reports describing rodent bladder pain models without overt bladder inflammation [28] or where bladder pain is not correlated with bladder inflammation [29]. These models, including the one reported here, may mimic more closely the clinical picture observed with Interstitial Cystitis/Painful Bladder Syndrome, especially since 90% of IC/PBS patients show no sign of active bladder inflammation but experience significant pain [30].

How PAR1 or PAR4 receptors produce hypersensitivity is not fully understood. Peripheral PAR4 activation increases calcitonin gene-related peptide (CGRP) and phopho-ERK1/2 immunoreactivity in dorsal root ganglion (DRG) neurons [31] and results in sensitization in models of joint pain [32, 33]. Since PAR4 receptors are found on bladder afferent neurons [17, 27], our findings may be due to direct PAR4 receptor activation of bladder afferents leading to hypersensitivity after intravesical PAR administration. A similar process may occur in IC/PBS patients that show elevated urine protease levels [34, 35], possibly resulting in greater intravesical PAR activation, and in turn, bladder hypersensitivity.

However, MIF inhibition with ISO-1 prevented bladder PAR1 or PAR4 receptor-induced hypersensitivity. Thus, our findings suggest that MIF and MIF receptors play a role in the development of bladder pain independent of direct PAR activation of bladder afferents. MIF has been recently implicated in other models of pain. Intrathecal injection of recombinant MIF in mice causes nociceptive behaviors, whereas spinal MIF inhibition with ISO-1 or an anti-MIF monoclonal antibody prevents the hypersensitivity associated with nerve constriction injury [1]. In addition, MIF^-/-^ mice do not develop hypersensitivity in nerve-injury pain models [2]. Finally, peripheral administration of recombinant MIF induces pain equivalent to that reported for models of pain induced with nerve injury or complete Freund’s adjuvant [2]. In this study, we used systemic administration of a MIF antagonist and further studies are needed to determine whether the site of inhibition is peripheral at the bladder or central in the spinal cord.

Intravesical PAR4-AP exposure increased expression of bladder MIF receptors CD74 and CXCR4, an effect blocked by MIF inhibition. Thus, since intravesical PAR4-AP induced bladder pain and bladder MIF receptor upregulation and both of these effects were prevented by the MIF antagonist, this argues for a peripheral site of action (i.e. bladder). Therefore, our results suggest that MIF mediates PAR-induced bladder pain acting, at least partly, through signal transduction mechanisms at the bladder, that are mediated partly through MIF-CXCR4 receptor interactions. The contribution of other MIF receptors may also be important in this process.

## Conclusions

Current animal models rely on producing pain secondary to an ongoing inflammatory insult [7, 22, 36, 37]. Our results suggest that intravesical PAR4 and PAR1 activation is a novel model of bladder pain without overtly causing bladder injury. Prevention of PAR-induced hypersensitivity by MIF antagonism suggests a strong link between bladder PAR receptor activity, urothelial MIF release, MIF-receptor mediated signaling in the bladder and abdominal hypersensitivity. Furthermore, MIF mediates PAR-induced bladder pain partially through bladder CXCR4 receptors. Future studies using mice deficient in urothelial MIF should provide new insights into the role of urothelial MIF in bladder pain and inflammation.
